# Early Pathogen Recognition and Antioxidant System Activation Contributes to *Actinidia arguta* Tolerance Against *Pseudomonas syringae* Pathovars *actinidiae* and *actinidifoliorum*

**DOI:** 10.3389/fpls.2020.01022

**Published:** 2020-07-22

**Authors:** M. Nunes da Silva, M. W. Vasconcelos, M. Gaspar, G. M. Balestra, A. Mazzaglia, Susana M. P. Carvalho

**Affiliations:** ^1^ GreenUPorto—Research Centre on Sustainable Agrifood Production, Faculty of Sciences, University of Porto, Vairão, Portugal; ^2^ CBQF—Centro de Biotecnologia e Química Fina—Laboratório Associado, Escola Superior de Biotecnologia, Universidade Católica Portuguesa, Porto, Portugal; ^3^ Dipartimento di Scienze Agrarie e Forestali, Università degli Studi della Tuscia, Viterbo, Italy

**Keywords:** *Actinidia chinensis*, antioxidant system, gene expression, *Pseudomonas syringae* pv. *actinidiae*, *Pseudomonas syringe* pv. *actinidifoliorum*, susceptibility

## Abstract

*Actinidia chinensis* and *A. arguta* have distinct tolerances to *Pseudomonas syringae* pv. *actinidiae* (Psa), but the reasons underlying the inter-specific variation remain unclear. This study aimed to integrate the metabolic and molecular responses of these two kiwifruit species against the highly pathogenic Psa and the less pathogenic *P. syringae* pv. *actinidifoliorum* (Pfm) bacterial strains. Disease development was monitored weekly till 21 days post inoculation (dpi), analysing a broad number and variety of parameters including: colony forming units (CFU), foliar symptoms, total chlorophylls, lipid peroxidation, soluble polyphenols, lignin and defense-related gene expression. At the end of the experimental period *A. chinensis* inoculated with Psa presented the highest endophytic bacterial population, whereas *A. arguta* inoculated with Pfm showed the lowest values, also resulting in a lower extent of leaf symptoms. Metabolic responses to infection were also more pronounced in *A. chinensis* with decreased total chlorophylls (up to 55%) and increased lipid peroxidation (up to 53%), compared with non-inoculated plants. Moreover, at 14 dpi soluble polyphenols and lignin concentrations were significantly higher (112 and 26%, respectively) in Psa-inoculated plants than in controls, while in *A. arguta* no significant changes were observed in those metabolic responses, except for lignin concentration which was, in general, significantly higher in Psa-inoculated plants (by at least 22%), comparing with control and Pfm-inoculated plants. Genes encoding antioxidant enzymes (*SOD*, *APX* and *CAT*) were upregulated at an earlier stage in Psa-inoculated *A. arguta* than in *A. chinensis*. In contrast, genes related with phenylpropanoids (*LOX1*) and ethylene (*SAM*) pathways were downregulated in *A. arguta*, but upregulated in *A. chinensis* in the later phases of infection. Expression of *Pto3*, responsible for pathogen recognition, occurred 2 dpi in *A. arguta*, but only 14 dpi in *A. chinensis*. In conclusion, we found that *A. arguta* is more tolerant to Psa and Pfm infection than *A. chinensis* and its primary and secondary metabolism is less impacted. *A. arguta* higher tolerance seems to be related with early pathogen recognition, the activation of plant antioxidant system, and to the suppression of ET and JA pathways from an earlier moment after infection.

## Introduction

*Pseudomonas syringae* pv. *actinidiae* (Psa) is a gram-negative bacterium that infects several *Actinidia* species, being responsible for the kiwifruit bacterial canker (KBC) (Vanneste et al., 2014; Kisaki et al., 2018). Following the first outbreaks of Psa in *A. chinensis* var. *deliciosa* cv. ‘Hayward’ in Japan during the 1980s, the disease spread to other important kiwifruit producing countries, such as South Korea and China (Serizawa et al., 1989; Koh, 1994; Cheng et al., 1995). Thereafter, epidemic outbreaks associated with a more pathogenic Psa strain were reported for the first time in several *A. chinensis* var. *chinensis* orchards located in South Korea (in 2006) and Italy (in 2008) (Ferrante and Scortichini, 2009; Koh et al., 2010). From then on, Psa quickly disseminated to several countries where kiwifruit production is highly relevant (such as, New Zealand, France, Portugal, Spain, Switzerland) rendering KBC a worldwide epidemic (European and Mediterranean Plant Protection Organization, 2018). KBC symptoms include leaf spotting, cane dieback, canker formation and fruit shrivelling, resulting in significant economic losses to the kiwifruit industry (Vanneste, 2017).

The pathovar *actinidiae* constitutes a genetically diverse group of bacteria with the capacity to infect several *Actinidia* spp., especially regarding genes responsible for type III effectors and phytotoxin production (Chapman et al., 2012). From the six Psa biovars identified until now, biovar 3, also known as Psa3 or Psa-V, seems to be the most pathogenic and the most widely disseminated, having been identified in Europe, New Zealand, Chile and China (Chapman et al., 2012; Vanneste et al., 2013; Dreo et al., 2014). Due to its rapid worldwide distribution, Psa3 is now considered a pandemic pathogen and has been included in the list for quarantine pests of the European and Mediterranean Plant Protection Organization (EPPO, 2014). Contrarily, the genetically close bacterial strain *P. syringae* pv. *actinidifoliorum* (Pfm), formerly known as Psa biovar 4 or Psa-LV (Cunty et al., 2015), is a less pathogenic population present in New Zealand, Australia and France (Chapman et al., 2012; Scortichini et al., 2012; Cunty et al., 2015). Whereas Psa3 is able to induce the formation of cankers in stems and conductive branches, compromising the vascular system of the infected plants, Pfm is characterized by its low severity, with the infection not progressing beyond foliar necrotic spots, and not causing important economic and production losses (Scortichini et al., 2012; Vanneste et al., 2013; Cunty et al., 2015). However, although their effector repertoire has been under analysis during the last decade with distinct bacterial virulence being attributed to differences in effector patterns, the full understanding on how bacteria are able to penetrate plant tissue and how plants activate their defense mechanisms is far from being achieved.

Generally, all plants of the genus *Actinidia* are affected by Psa, but there seems to be considerable variation in their tolerance to this pathogen. Field evidence has shown that cultivars of *A. chinensis* var. *chinensis*, such as ‘Hort16A’ and ‘Jin Tao’, present high susceptibility to Psa compared to cultivars of *A. chinensis* var. *deliciosa*, such as ‘Hayward’ (Balestra et al., 2009). In addition, *A. arguta*, *A. macrosperma*, *A. polygama* and *A. rufa* were classified as tolerant to Psa (Nardozza et al., 2015; Kisaki et al., 2018; Michelotti et al., 2018), but the molecular and biochemical mechanisms responsible for this tolerance are not fully known (Wang et al., 2018; Nunes da Silva et al., 2019).

The basal plant resistance mechanisms, also called innate immunity, are the first line of defense against various pathogens, including bacteria, and are triggered *via* plant cells pattern recognition receptors, which recognize specific pathogen-associated molecular patterns characteristic to each pathogen (Wurms et al., 2017a; Michelotti et al., 2018; Wang et al., 2018). As such, microorganism recognition by host plants involves a complex physiological and molecular reprogramming that encompasses overregulation of pathogenesis-related genes (PR genes) and defense-related proteins (Michelotti et al., 2018). If a pathogen is able to overcome basal defenses, plants may respond with a secondary line of defense, the hypersensitive response (HR), in which plants can become tolerant to a wide range of pathogens over a long period of time in a phenomenon called systemic acquired resistance (SAR) (Cellini et al., 2014). This phenomenon includes changes in structural defenses, such as strengthening of the cell wall through the deposition of callus and lignin, increased enzymatic activity, and overexpression of the aforementioned PR-genes (Michelotti et al., 2018).

Plant defense against pathogens is also associated with molecular networks based on the activity of reactive oxygen species (ROS), such as superoxide (O_2_^−^) and hydrogen peroxide (H_2_O_2_), and phytohormone signalling, including jasmonic acid (JA) and salicylic acid (SA) (Petriccione et al., 2014; Wurms et al., 2017b). Increased production of ROS during stress, in particular, may pose a threat to cell homeostasis due to subsequent lipid peroxidation, protein oxidation, nucleic acid damage, and enzyme inhibition, ultimately resulting in cell death (Sharma et al., 2012). Therefore, in order to avoid oxidative damage, plants synthetize antioxidant enzymes directly involved in ROS detoxification, such as superoxide dismutase (SOD), catalase (CAT), and ascorbate peroxidase (APX). Along with antioxidant enzymes, phenolic compounds are among the most important defense-related molecules produced by plants, and include flavonoids, anthocyanins, phytoalexins, tannins and lignin (Lattanzio, 2013). Miao et al. (2009) compared phenolic compounds content in shoots and leaves of kiwifruit cultivars with different resistance to KBC and observed that prior to inoculation phenolic compounds in tolerant cultivars were significantly higher than in susceptible ones, and that after inoculation their concentration increased in both tolerant and susceptible cultivars, suggesting that this metabolic pathway may play an important role in plant defense.

Lignin, a complex phenolic polymer, is known to accumulate in plant tissues after infection by pathogenic fungi or bacteria, acting by diminishing the mechanical pressure resulting from pathogen penetration and reproduction (Gallego-Giraldo et al., 2018). Inoculation of *Brassica rapa* with *Erwinia carotovora*, for example, induced the activation of genes that regulate lignin biosynthesis, leading to increased concentration of p-coumaryl, one of the three lignin monolignols, and to a 43% increase in lignin concentration in plant tissues just 3 days post inoculation (dpi) (Zhang et al., 2007). Despite being key players in plant defense against pathogens, how phenolic compounds are regulated in Psa- and Pfm-infected kiwifruit plants is still poorly understood. It is clear that the interaction between kiwifruit plants and Psa and Pfm is highly complex, with multiple bacterial factors and signalling events occurring simultaneously in the host plant tissues, which ultimately defines the susceptibility or tolerance of the plant exposed to the pathogen (Petriccione et al., 2015; Wurms et al., 2017a; Wang et al., 2018; Tahir et al., 2019).

In a preliminary study, focused on the short-term analysis of the expression of key defense-related genes, we have found that *A. arguta* cv. ‘Ken’s Red’ is more tolerant than *A. chinensis* to Psa and Pfm infection by limiting bacterial endophytic population through inhibition of JA and ethylene (ET) pathways (Nunes da Silva et al., 2019). However, that study was limited to the early stages of the plant infection (up to 5 dpi) and only addressed the expression of plant-defense related genes, limiting the possibilities of interpretation. Although it has been suggested that the antioxidant system, ET and SA regulatory pathways and pathogen recognition mechanisms play important roles in plant defense against Psa (Petriccione et al., 2013; Reglinski et al., 2013; Cellini et al., 2014; Petriccione et al., 2014; Nunes da Silva et al., 2019), information on how these networks are activated and regulated and how they differ between tolerant and susceptible kiwifruit plants is still very scarce. This greatly hinders the possibility to identify new metabolic and/or genotypic traits that could be used as markers for kiwifruit plant breeding and for identifying novel plant protection strategies.

The aim of this work was to analyze the dynamics of Actinidia/Psa and Actinidia/Pfm pathosystems, in order to identify and integrate key metabolic or genotypic traits that account for the higher tolerance of some *Actinidia* spp. To that end, we evaluated weekly (for 21 days post inoculation with Psa and Pfm) how two kiwifruit species with reported distinct tolerance to KBC (*A. chinensis* and *A. arguta*) responded to a large number and variety of parameters including: bacterial endophytic population in plant tissues, leaf symptoms, primary and secondary metabolism, antioxidant system and pathogen recognition.

## Methods

### Plant Maintenance and Inoculation

Micropropagated plants of *A. chinensis* var. *deliciosa* cv. ‘Hayward’ and *A. arguta* var. *arguta* cv. ‘Weiki’ were purchased from QualityPlant—Investigação e Produção em Biotecnologia Vegetal, Lda. (Castelo Branco, Portugal). A modified Murashige and Skoog (MS) agar medium was used for plant maintenance during the trial period, and consisted in sucrose (30 g l^−1^), myo-inositol (100 mg l^−1^), thiamine-HCl (1 mg l^−1^), nicotinic acid (1 mg l^−1^), pyridoxine (1 mg l^−1^), glycine (1 mg l^−1^) and benzylaminopurine (0.5 mg l^−1^), adjusted to pH 5.7 with KOH. Plants were kept in sets of three plants in 200 ml containers in a climate chamber (Aralab Fitoclima 5000EH, Aralab, Rio de Mouro, Portugal) with a 16 h day photoperiod and 200 µmol s^−1^ m^−2^ of photosynthetic photon flux density at plant level. Temperatures were set to 21°C during the light period and to 19°C during the dark period, and relative humidity was maintained at 80%.

A pathogenic Psa strain (CFBP7286, isolated in 2008 in Italy from *A. chinensis* var. *chinensis*) and a Pfm strain (CFBP18804, isolated in 2010 in New Zealand from *A. chinensis* var. *chinensis*) were grown for 48 h on nutrient agar with 5% sucrose (NSA) at 27°C in the dark. In the day of inoculation, a fresh 1–2 × 10^7^ CFU ml^−1^ inoculum was prepared in sterile Ringer’s solution (NaCl 0.72%, CaCl_2_ 0.017% and KCl 0.037%, pH 7.4).

A total of 135 plants (45 plants per treatment) were inoculated with one of the bacterial suspensions or with Ringer’s solution alone (control) by dipping plant shoots in the solution for 15 s. A set of parameters were evaluated 1, 2, 7, 14 and 21 days post inoculation (dpi), including: 1) endophytic bacterial population in plant tissues and leaf symptoms occurrence; 2) primary and secondary metabolism (total chlorophylls, soluble polyphenols, lignin, and reporter genes for the JA- and ET-biosynthetic pathways); 3) antioxidant system (lipid peroxidation, expression of genes *SOD*, *APX* and *CAT*); and 4) pathogen recognition (expression of gene *Pto3*, which interacts with pathogen virulence effectors and activates plant defense). The experiment was concluded at 21 dpi to allow the appearance of disease symptoms but also ensuring that plant tissues were still viable to guarantee a proper RNA extraction.

### Scoring of Foliar Symptoms and Sampling

Foliar symptoms were evaluated taking into account the percentage of leaf area affected by necrotic spots, according to the following scale: 0: no symptoms, healthy plant; I: <5% of the leaf area affected; II: 5–9% of affected leaf area; III: 10–14% of affected leaf area; IV: 15–19% of affected leaf area; V: >20% of affected leaf area (adapted from Cellini et al., 2014). Sampling was performed in each time-point of analysis by removing plants from the culturing medium and cutting the tip (*ca*. 0.2 cm length) of every leaf with sterile scissors for CFU determination. The remaining plant was flash-frozen in liquid nitrogen, macerated with mortar and pestle and stored at −80°C for metabolites and gene expression analysis. Each biological replicate was obtained by pooling three plants from the same container and three independent biological replicates were analyzed per treatment and time-point.

### Endophytic Bacterial Population

Estimation of Psa and Pfm colony forming units (CFU) in plant tissues was performed using an adapted method from Cellini et al. (2014). Samples were surface sterilized by washing in 70% ethanol for 1 min, followed by a 1-min treatment with 1% sodium hypochlorite, after which they were rinsed twice in sterile water for 1 min. After maceration in 10 ml Ringer’s solution, samples were sequentially diluted ten-fold up to 10^−4^, and three replicates of 100 μl from each ten-fold dilution were plated on NSA medium. After plate incubation at 27°C for 48 h in the dark, the number of colonies in each plate were counted and CFU were estimated taking into consideration the fresh weight of each sample.

### Malondialdehyde

An adapted protocol from Li (2000) was used for malondialdehyde (MDA) quantification. Fifty milligrams of plant sample were added to 500 µl of 0.1% trichloroacetic acid (w/v) and mixed vigorously for 90 s. After sample centrifugation for 5 min at 10,000*g*, 250 µl of the supernatant were transferred to a new microcentrifuge tube and mixed with 1 ml of 0.5% thiobarbituric acid in 20% trichloroacetic acid. The mixture was incubated at 100°C for 30 min, after which the reaction was stopped by rapidly transferring the samples to ice. Samples were centrifuged at 10,000*g* for 10 min and the supernatant was used to measure absorbances spectrophotometrically at 532 and 600 nm in a nanophotometer (Implen GmbH, München, Germany). MDA (nmol g^−1^ fresh weight) was determined by the following formula:

$$MDA=\frac{(Abs_{532}-Abs_{600})\times volume}{\epsilon =155mM/cm\times biomass}$$

### Primary and Secondary Metabolites

Lyophilized plant tissues (50 mg) were extracted with 1.5 ml of 80% aqueous methanol (v/v) in an ultrasonic bath for 30 min. Samples were centrifuged for 15 min at 15,000*g* and the supernatant was transferred to a new microcentrifuge tube, which was kept on ice during the analysis. The methanolic extract was used for total chlorophyll and total soluble polyphenols quantification. The solid biomass that remained in the original microcentrifuge tube was successively extracted with water, acetone and hexane in the conditions previously described, after which it was dried at 70°C for 48 h and used for lignin quantification.

Total chlorophyll was quantified as in Sumanta et al. (2014) by recording samples absorbances at 470, 652 and 665 nm in a nanophotometer (Implen GmbH, München, Germany). Pigments (μg g^−1^ dry weight) were quantified as:

$$Chlorophyll\;a=(16.72Abs_{665}-9.16Abs_{652})\times volume/biomass$$

$$Chlorophyll\;b=(34.09Abs_{652}-15.28Abs_{665})\times volume/biomass$$

$$Carotenoids=(1000Abs_{470}-1.63Chla-104.96Chlb)/(221\times biomass)$$

Total soluble polyphenols were quantified according to the Folin method adapted from Marinova et al. (2005). To 100 µl of the methanolic extract, 4.5 ml of ultrapure water and 500 µl of Folin–Denis’ reagent were added. The reaction was allowed to occur for 5 min at RT, after which 5 ml of 7% sodium carbonate were added. After incubation for 1 h at RT in the dark, 2 ml of ultrapure water were added. Sample absorbance was recorded at 750 nm and total soluble polyphenols were determined in each sample through a gallic acid standard curve.

Lignin quantification was performed through an adapted method from Hatfield et al. (1999). Ten milligrams of dried sample obtained as previously described were mixed with 1 ml of 12.5% acetyl bromide (in acetic acid, v/v) and incubated for 1 h at 50°C with vigorous stirring. After centrifugation for 5 min at 15,000*g*, 100 μl of sample were transferred to a new microcentrifuge tube with 200 µl of acetic acid and 150 µl of 0.3 M sodium hydroxide. After adding 50 µl of 0.5 M hydroxylamine hydrochloride and 500 µl of acetic acid, sample absorbance was recorded at 280 nm in a nanophotometer (Implen GmbH, München, Germany) and a lignin calibration curve was used for soluble lignin estimation in each sample.

### Gene Expression

Total RNA was extracted according to an adapted protocol from Cellini et al. (2014). After tissue homogenization with liquid nitrogen, 1 ml of warm (70°C) extraction buffer (100 mM Tris–HCl, pH 8.0, cetyltrimethylammonium bromide 4% w/v, polyvinylpyrrolidone K40 4% w/v, 30 mM ethylenediamine tetraacetic acid, 2.0 M NaCl, spermidine 0.1% w/v, *β*-mercaptoethanol 2% v/v) was added to *ca*. 200 mg of sample. Samples were mixed vigorously and incubated at 70°C for 10 min. Subsequently, 1 ml of chloroform-isoamyl alcohol (24:1, v/v) was added, and samples were centrifuged for 15 min at 15,000*g*. The upper phase was collected to a new tube, washed with chloroform-isoamyl alcohol (24:1, v/v), centrifuged for 15 min at 15,000*g* and the supernatant combined with 250 µl of 12 M LiCl by gentle pipetting. Samples were incubated overnight at −20°C, after which they were centrifuged at 15,000*g* for 35 min at 4°C. The supernatant was discarded and the pellet was washed in cold 70% ethanol, dried, and resuspended in 50 μl sterile DEPC water. Single-stranded cDNA was synthesized using iScript cDNA Synthesis Kit (Bio-Rad, California, USA) according to the manufacturer’s instructions in a Doppio Thermal Cycler (VWR, Oud-Heverlee, Belgium).

Primers for *LOX1*, *SAM* and *Pto3* were designed using Primer3 for an expected PCR product of 100–200 bp and primer annealing temperatures between 56 and 58°C, whereas primer sequences for *ACT* were obtained from Ledger et al. (2010), *PP2A* from Nardozza et al. (2013) and *APX*, *CAT* and *SOD* from Petriccione et al. (2015). Reverse transcription polymerase chain reactions (qRT-PCR) were performed on a StepOne Real-Time PCR System (Applied Biosystems, California, USA) with the following reaction conditions: 3 min at 95°C and 40 cycles with: 15 s at 95°C, 30 s at each primer pair optimal annealing temperature (**Table 1**) and 30 s at 72°C. Amplifications were carried out using a final volume of 20 µl which consisted of 1 µl of the specific primers at 6 µM, 10 µl of 2× iQ SYBR Green Supermix (Bio-Rad, California, USA) and 8 µl of a 1:50 dilution of the template cDNA. Melt curve profiles were analyzed for each tested gene. The comparative CT method (ΔΔCT, Livak and Schmittgen, 2001) was used for the relative quantification of gene expression values using actin (*ACT*) and protein phosphatase 2A (*PP2A*) genes as control transcript (Petriccione et al., 2015) and the plants inoculated with Ringer’s solution (non-inoculated controls) as the reference sample. For each biological replicate and target gene two technical replicates were analyzed.

**Table 1:** Primer sequences (Forward—F and Reverse—R) and annealing temperature of reference genes (RG) and target genes used for RT-qPCR analysis.

Gene *(Accession number)*	Name	Primer sequence (5′-3′)		T_ann_ (°C)	Reference
***ACT*** *FG440519*	**Actin** (RG)			56.0	Ledger et al. (2010)
	F	CCAAGGCCAACAGAGAGAAG			
	R	GACGGAGGATAGCATGAGGA			
***PP2A*** *FG522516*	**Protein phosphatase 2A** (RG)			55.2	Nardozza et al. (2013)
	F	GCAGCACATAATTCCACAGG			
	R	TTTCTGAGCCCATAACAGGAG			
***APX*** *FG408540*	**Ascorbate peroxidase**			57.8	Petriccione et al. (2015)
	F	GGAGCCGATCAAGGAACAGT			
	R	AACGGAATATCAGGGCCTCC			
***CAT*** *FG470670*	**Catalase**			56.9	
	F	GCTTGGACCCAACTATCTGC			
	R	TTGACCTCCTCATCCCTGTG			
***SOD*** *FG471220*	**Superoxide dismutase**			57.8	
	F	CACAAGAAGCACCACCAGAC			
	R	TCTGCAATTTGACGACGGTG			
***LOX1*** *DQ497792*	**Lipoxygenase 1**			53.5	This study
	F	GTTAGAGGGGTGGTGACTCT			
	R	CTTTAGCACTGCTTGGTTGC			
***SAM*** *U17240*	**S-adenosylmethionine synthetase**			55.1	
	F	GAATAGTACTTGCCCCTGGC			
	R	TACAAATCGACCAGAGGGGT			
***Pto3*** *KR054654*	**Pto-like protein kinase 3**			56.6	
	F	TACCACTGTCGCCATTAAGC			
	R	CCAGCGAACCATTGACCATA			

T_ann_, annealing temperature.

### Statistical Analysis

Data were analyzed with GraphPad Prism version 6.0 (GraphPad Software, Inc., California, USA). Significant differences between treatments were determined by analysis of variance (ANOVA) followed by Fisher’s LSD test (*p <*0.05). For CFU and relative gene expression analysis mean comparison was carried out within the same kiwifruit species (i.e. having the time-point and the two *P. syringae* pathovars as factors), whereas for total chlorophylls, MDA, soluble polyphenols and lignin concentrations significant differences among treatments were analyzed within each time-point.

## Results

### Disease Symptoms and Psa Endophytic Population in Plant Tissues

**Figure 1:**
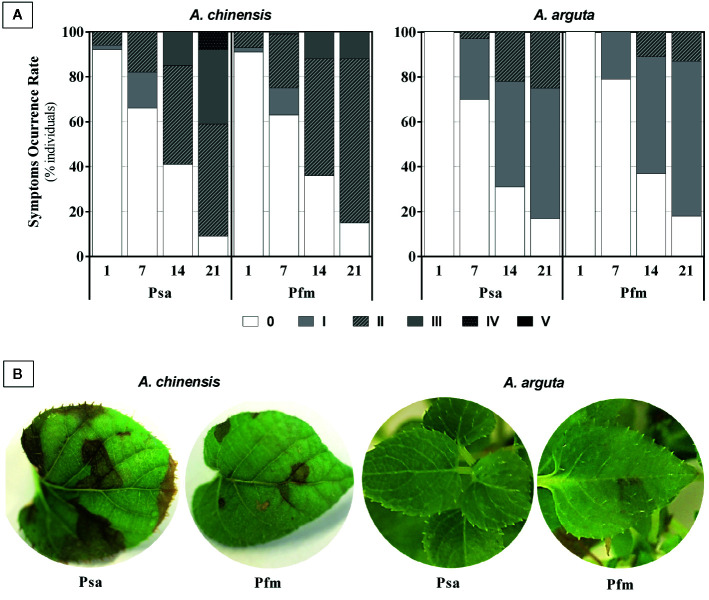
**(A)** Symptom occurrence rate (percentage of individuals showing disease symptoms) in *Actinidia chinensis* var. *deliciosa* cv. ‘Hayward’ and *A. arguta* var. *arguta* cv. ‘Weiki’ plants inoculated with *Pseudomonas syringae* pv. *actinidiae* (Psa) or *P. syringae* pv. *actinidifoliorum* (Pfm) registered 1, 7, 14 and 21 days post inoculation (dpi). Foliar symptoms were scored taking into account the percentage of leaf area affected by necrotic spots: 0: no symptoms; I: <5%; II: 5–9%; III: 10–14%; IV: 15–19%, V: >20%. **(B)** Most typical foliar symptoms observed 21 dpi in both kiwifruit species inoculated with Psa or Pfm.

Mock-inoculated *A. chinensis* and *A. arguta* plants (plants inoculated with saline solution without bacteria) did not show visual symptomatology throughout the experimental period. On the contrary, *A. chinensis* plants began to show foliar necrosis as soon as 1 day post inoculation (dpi), whereas in *A. arguta* foliar symptoms started to appear 7 dpi, regardless of the bacterial strain (**Figure 1A**). The percentage of *A.* *arguta* plants showing leaf necrotic spots was always smaller than in *A. chinensis*. In fact, in this species, at 21 dpi, 91% of plants had symptoms of Psa infection, with 41% of the plants having leaf symptoms of grade III or grade IV, whereas in *A. arguta* 83% of Psa-inoculated plants had necrotic spots, but these did not progress beyond grade II (**Figure 1A**). In Pfm-inoculated plants, disease symptoms were slightly milder than in Psa-infected plants, reaching grade III in *A. chinensis*, and only grade II in *A. arguta*. At 21 dpi, the most common symptoms in *A. chinensis* included pronounced dark-brown areas of necrotic tissues with varying diameter when inoculated with Psa and smaller light-brown necrotic areas (up to 5 mm) in Pfm-inoculated plants, whereas in *A. arguta* leaf spot appearance was rare and fainter (**Figure 1B**).

**Figure 2:**
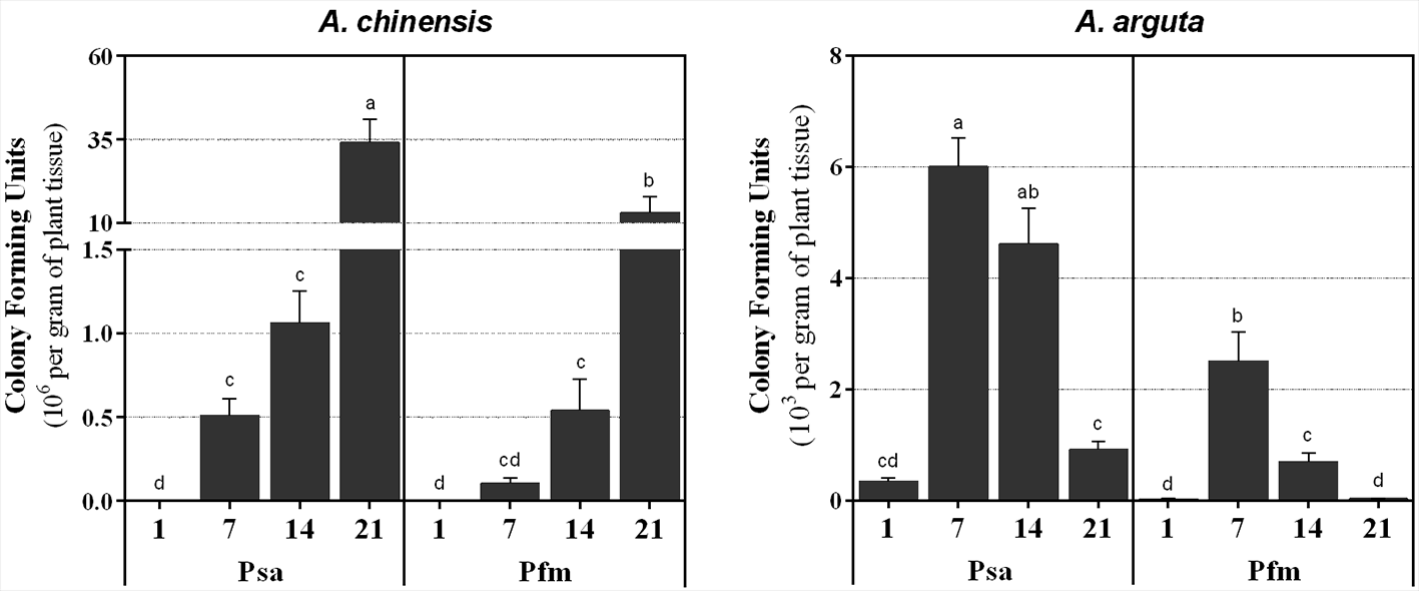
Number of colony forming units (CFU per gram of plant tissue) in *Actinidia chinensis* var. *deliciosa* cv. ‘Hayward’ and *A. arguta* var. *arguta* cv. ‘Weiki’ plants inoculated with *Pseudomonas syringae* pv. *actinidiae* (Psa) or *P. syringae* pv. *actinidifoliorum* (Pfm) registered 1, 7, 14 and 21 days post inoculation (dpi). For each kiwifruit species, columns represent the mean of three independent replicates (consisting in the pool of three plants each) ± standard error. Columns with different letters are significantly different at *p <*0.05. Note that the CFU scale (left x-axis) is different in the two panels.

In *A. chinensis*, a significant increase in bacterial colonization was observed throughout the experimental period (**Figure 2**), reaching a maximum level of 34.0 ± 7.0 × 10^6^ CFU g^−1^ at 21 dpi with Psa and 13.1 ± 4.8 × 10^6^ CFU g^−1^ with Pfm. Contrastingly, *A. arguta* Psa-inoculated plants presented a colonization peak at 7 dpi, with 6.0 ± 0.5 × 10^3^ CFU g^−1^ (**Figure 2**), after which CFU significantly decreased by 0.8-fold until reaching 0.9 ± 0.2 × 10^3^ CFU g^−1^ at 21 dpi. The evolution of endophytic bacterial populations was identical between Psa and Pfm, regardless of plant species, with Psa bacterial density within plant tissues being always higher than Pfm, up to 4.9-fold (7 dpi) in *A. chinensis* and 29.8-fold (21 dpi). in *A. arguta*.

### Primary and Secondary Metabolites

**Figure 3:**
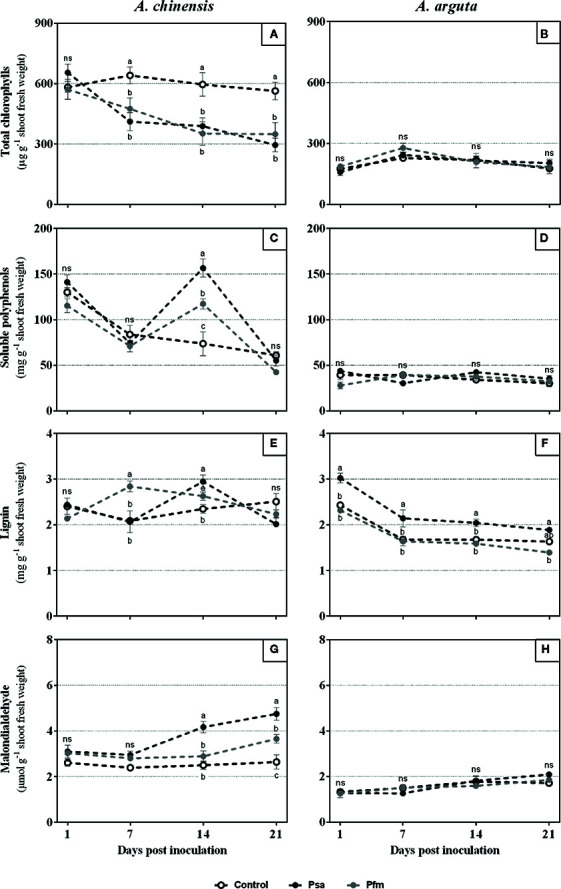
Total chlorophylls **(A, B)**, soluble polyphenols **(C, D)**, lignin **(E, F)** and malondialdehyde **(G, H)** concentrations in *Actinidia chinensis* var. *deliciosa* cv. ‘Hayward’ and *A. arguta* var. *arguta* cv. ‘Weiki’ plants inoculated with *Pseudomonas syringae* pv. *actinidiae* (Psa) or *P. syringae* pv. *actinidifoliorum* (Pfm) registered 1, 7, 14 and 21 days post inoculation (dpi). Symbols represent the mean of three independent replicates (consisting in the pool of three plants each) ± standard error. Within each time-point, symbols with different letters are significantly different at p < 0.05 (“ns” indicates no significant differences).

*A. chinensis* inoculation led to a significant decrease in total chlorophyll concentration as soon as 7 dpi, regardless of the bacterial strain (**Figure 3A**). At the end of the experimental period, Psa and Pfm inoculation induced, respectively, 55 and 39% of chlorophyll loss comparing with the initial values (1 dpi). Contrastingly, no significant alterations were found in total chlorophyll concentration in *A. arguta*-inoculated plants throughout the experimental period (**Figure 3B**).

Total soluble polyphenols were only significantly different among inoculated and control plants of *A. chinensis* at 14 dpi, where Psa-inoculated plants had a significantly higher concentration than Pfm (33%) and control plants (112%), with 156.4 ± 10.0 mg g^−1^ (**Figure 3C**). At the end of the experimental period total soluble polyphenols were up to 63% lower in all treatments, averaging 53 mg g^−1^, comparing with 1 dpi. Contrastingly, once more no significant differences were observed in soluble polyphenols in *A. arguta*, when comparing control and inoculated plants (**Figure 3D**).

Concerning lignin concentration, it was found that *A. chinensis* Pfm-inoculated plants had a significantly higher value at 7 dpi (36%) and 14 dpi (12%) when compared with control plants (**Figure 3E**). In *A. chinensis* Psa-inoculated plants, lignin concentration only differed from control plants at 14 dpi, being 26% higher. In *A. arguta*, contrastingly to what was observed for the other parameters analyzed, a significant variation in lignin concentration occurred between different treatments throughout the experimental period (**Figure 3F**). One dpi, Psa-inoculated plants presented 3.0 ± 0.1 mg g^−1^ lignin, which was 24 and 31% higher than control and Pfm-inoculated plants, respectively. After this time-point, lignin concentration decreased in all treatments and, in general, Psa-inoculated plants remained with a significantly higher lignin concentration compared with control and Pfm-inoculated plants (up to 14%).

MDA concentration (**Figure 3G**) significantly increased after 14 dpi in Psa- and 21 dpi in Pfm-inoculated *A. chinensis* plants when compared with control plants. By the end of the experimental period, MDA concentration was 53 and 23% higher in Psa- and Pfm-inoculated plants, respectively, resulting in 4.8 ± 0.3 and 3.7 ± 0.2 µmol g^−1^ MDA, whereas in control plants this value was significantly lower (2.6 ± 0.3 µmol g^−1^ MDA). Similarly to what was observed for total chlorophyll concentration, in *A. arguta* MDA concentration did not differ significantly in inoculated and control plants (**Figure 3H**).

### Gene Expression

**Figure 4:**
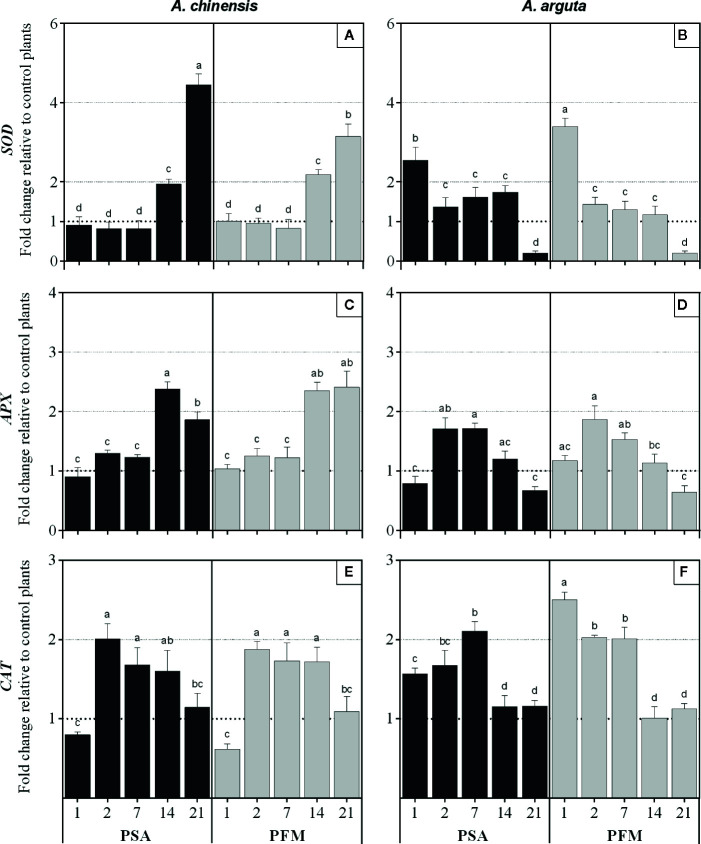
Relative fold of expression of *SOD* (superoxide dismutase), *APX* (ascorbate peroxidase) and *CAT *(catalase) genes in *Actinidia chinensis* var. *deliciosa* cv. ‘Hayward' (panels **A**, **C** and **E**, respectively) and in *A. arguta* var. *arguta* cv. ‘Weiki’ plants (panels **B**, **D** and **F**, respectively) inoculated with *Pseudomonas syringae* pv. *actinidiae* (Psa) or *P. syringae* pv. *actinidifoliorum* (Pfm) registered 1, 2, 7, 14 and 21 days post inoculation (dpi). Columns represent the mean of three independent replicates (consisting in the pool of three plants each) ± standard error. Within the same kiwifruit species, columns with different letters are significantly different at p < 0.05. One-fold change represents no relative change in gene expression, as compared with non-inoculated control plants.

In *A. chinensis* infected plants, *SOD* relative expression only started to be significantly upregulated at 21 (Psa) and 14 dpi (Pfm), reaching a 4.5- and a 3.1-fold increase (respectively) compared to mock-inoculated control plants (**Figure 4A**). On the other hand, in *A. arguta SOD* overexpression occurred as soon as 1 dpi and it significantly decreased from 1 to 2 dpi, by 0.46-fold in Psa-inoculated plants and by 0.58-fold in Pfm, being further downregulated by the end of the experimental period (**Figure 4B**). A similar trend was observed in *APX* regulation since *A. chinensis* infected plants only showed overexpression of this gene at 14 dpi, being 2.4-fold higher compared with control plants, regardless of the bacterial strain (**Figure 4C**). In *A. arguta*, plant inoculation induced *APX* overexpression already at 2 dpi (1.8-fold for Psa and 1.9-fold for Pfm), decreasing thereafter and becoming downregulated at 21 dpi (**Figure 4D**). In *A. chinensis* *CAT* overexpression was observed at 2 dpi in plants inoculated with both bacterial strains, decreasing until the end of the experimental period (**Figure 4E**). Contrastingly, in *A. arguta* *CAT* overexpression occurred as soon as 1 dpi, particularly in Pfm-inoculated plants, which reached a 2.5-fold increase relatively to control plants (**Figure 4F**). From 14 dpi on, *CAT* expression further decreased to basal levels in plants inoculated with either bacterial strain.

**Figure 5:**
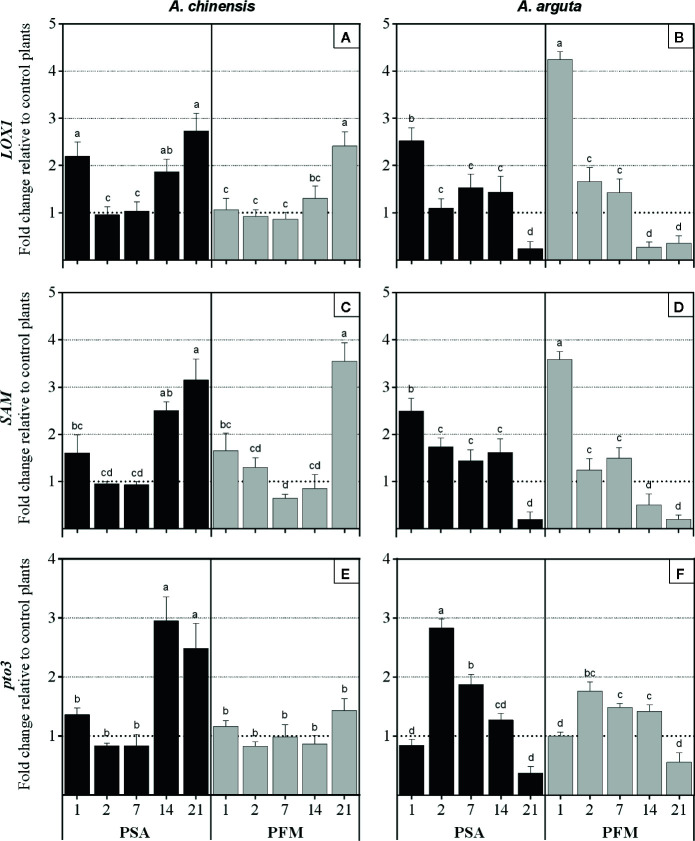
Relative fold of expression of *LOX1* (lipoxygenase 1), *SAM* (s-adenosylmethionine synthetase) and *Pto3* (Pto-like protein kinase 3) genes in *Actinidia chinensis* var. *deliciosa* cv. ‘Hayward' (panels **A**, **C** and **E**, respectively) and in *A. arguta* var. *arguta* cv. ‘Weiki’ plants (panels **B**, **D** and **F**, respectively) inoculated with *Pseudomonas syringae* pv. *actinidiae* (Psa) or *P. syringae* pv. *actinidifoliorum* (Pfm) registered 1, 2, 7, 14 and 21 days post inoculation (dpi). Columns represent the mean of three independent replicates (consisting in the pool of three plants each) ± standard error. Within the same kiwifruit species, columns with different letters are significantly different at p < 0.05. One-fold change represents no relative change in gene expression, as compared with non-inoculated control plants.

A similar trend in relative fold of expression throughout the experimental period was observed when comparing *LOX1* and *SAM*, within the same plant species, with little variation being observed between Psa- and Pfm-inoculated plants (**Figures 5A–D**). In *A. chinensis*, both transcripts were overexpressed in Psa-inoculated plants 1 dpi, but returned to basal values at 2 and 7 dpi, significantly increasing thereafter (**Figures 5A, C** **)**. After Pfm inoculation, *LOX1* and *SAM* relative expression remained unaltered until 21 dpi, where a 2.4- and 3.6-fold overexpression was observed, compared with non-inoculated plants. In *A. arguta*, *LOX1* and *SAM* relative fold of expression was increased 1 dpi, independently of the bacterial strain inoculated, significantly decreasing throughtpt out the experimental period, by *ca*. 0.94-fold in both inoculums, being inclusively downregulated at 14 and/or 21 dpi (**Figures 5B, D** **)**.

Finally, in *A. chinensis* the relative fold of expression *Pto3* was only significantly increased at 14 dpi in Psa-inoculated plants (becoming 3.0-fold higher than mock-inoculated controls) and remaining elevated until the end of the experimental period. Contrastingly, no significant alterations were observed in the expression of this gene in Pfm-inoculated *A. chinensis* plants (**Figure 5E**). In *A. arguta*, *Pto3* overexpression occurred at 2 dpi, being 3.3-fold in Psa- and 1.8-fold in Pfm-inoculated plants, gradually decreasing until the end of the experimental period (**Figure 5F**).

## Discussion

### Bacterial Progression Is More Restricted in *A. arguta*

Limited bacterial progression within plant tissues is regarded as a tolerance trait. However, until now very few long-term disease assessments under the same controlled environmental conditions have been performed to compare Psa- and Pfm-inoculated *A. chinensis* and *A. arguta* plants, hindering the full comprehension of bacterial infection and plant tolerance mechanisms (Wurms et al., 2017a). Datson et al. (2015) reported that in *A. arguta* plants stab-inoculated with a Psa strain, the endophytic bacterial population was lower and more restricted to the site near the inoculation zone when compared with *A. chinensis*, even four weeks after inoculation. Moreover, in a previous short-term study, we observed that bacterial density of not only Psa but also Pfm inside plant tissues was lower in *A. arguta* than in *A. chinensis* up to 5 days post inoculation (Nunes da Silva et al., 2019).

In the present work, throughout all experimental period the evolution of Psa endophytic population seemed to accompany the appearance of foliar symptoms, with a rapid and intense bacterial multiplication in *A. chinensis* tissues, especially after inoculation with Psa, whereas in *A. arguta* tissues showed lower bacterial density and symptom severity (**Figures 1** and **2**). Together, these results confirm the higher susceptibility of *A. chinensis* probably because bacteria are able to easily progress and reproduce inside its vascular system, whereas in *A. arguta* bacterial movement and reproduction is more restricted (Wang et al., 2017). It is interesting to note that in the present work foliar symptoms began to appear as soon 1 dpi, whereas in other studies symptoms only appear after the first 5 dpi (McAtee et al., 2018). We hypothesize that the inoculation method selected in the current work (i.e. plant dipping in inoculum) favored the accumulation of bacterial droplets in plant leaves, propelling the entrance of bacteria into plant tissue and the appearance of disease symptoms shortly after infection. Psa population in plant tissues were always higher than Pfm’s, thus demonstrating the less pathogenic character of the second bacterial strain, even when inoculated into the more susceptible kiwifruit species. Looking only at stems mean lesion length and not to bacterial density in plant tissues *per se*, Wurms et al. (2017a) compared *A. chinensis* var. *deliciosa* cv. ‘Hayward’ plants inoculated with Psa or Pfm, and observed that Psa infection induced more severe symptoms, with the appearance of dark-brown necrotic tissues and water-soaked appearance near the site of inoculation. The present work confirms these findings, as in both plant species leaf symptoms were less severe following Pfm infection, with light-brown necrotic areas appearing after Psa inoculation and almost no necrotic tissues visible after Pfm inoculation. Our results attest for the higher pathogenicity of Psa strains belonging to biovar 3, as compared to Pfm, as previously suggested (Hoyte et al., 2015).

### Primary and Secondary Metabolism Is Less Impacted in *A. arguta* Than in *A. chinensis*

Plant infection by bacterial pathogens frequently leads to loss of chloroplast functions and, consequently, to tissue chlorosis or necrosis, impairing plant fitness and propelling disease (Lu and Yao, 2018). Transcripts related with photosynthesis were reported to be repressed in susceptible *Actinidia chinensis* var. *chinensis* cultivars, but highly expressed in the more tolerant *A. eriantha* species after Psa infection (Wang et al., 2017). Here, decreased total chlorophyll concentration was observed in *A. chinensis* after infection with Psa and Pfm, whereas in *A. arguta* inoculated with either bacterial strain no significant alterations in total chlorophyll concentration over the experimental period were recorded (**Figure 3**). Loss of these photosynthetic pigments in *A. chinensis* is probably related to the higher prevalence of tissue necrosis observed (**Figure 1**), thus demonstrating the more tolerant character of *A. arguta*. Concerning phenolic compounds, increased polyphenols concentration was observed in *A. chinensis* 14 dpi with Psa and Pfm, probably as part of the defense mechanisms employed by the infected plants (**Figure 3**). Contrastingly, no significant alterations were observed in soluble polyphenols in *A. arguta*, which is in line with what was observed for total chlorophylls and MDA concentrations, demonstrating that inoculation did not induce severe alterations in plant metabolism. Lignin concentration increased 7 dpi in *A. chinensis* Pfm-inoculated plants and 14 dpi in Psa-inoculated ones, whereas in *A. arguta* it was significantly higher in Psa-inoculated plants in all time-points analyzed. So far, the role of lignin accumulation in Psa- and Pfm-infected kiwifruit plants has not been properly explored, but we hypothesize that increased lignin concentration in Psa-inoculated plants may be one of the defense mechanisms employed by *A. arguta* to restrain pathogen penetration and/or migration within plant tissues, possibly contributing to its higher tolerance to Psa.

*LOX1* and *SAM* are involved in JA and ET biosynthesis pathways. Whereas *LOX1* converts α-linolenic acid into 13-hydroperoxyoctadecatrienoic acid in the jasmonic acid biosynthesis, *SAM* encodes S-adenosylmethionine synthetase, a precursor of ethylene biosynthesis (Kolomiets et al., 2000). Although SAM is the precursor of not only ET but also of polyamines, due to its key regulatory role during the first step of ET biosynthesis it is frequently used as reporter for ET metabolism (Zhao et al., 2017). Genes involved in ET biosynthesis, such as *AP2*/*ERF*, *EIN2* and *SAM*, were found to be upregulated just 2 dpi in *A. chinensis* plants inoculated with Psa (Wurms et al., 2017a; Wurms et al., 2017b, Nunes da Silva et al., 2019). *LOX1* increased activity was also previously reported in *A. chinensis* and *A. arguta* Psa- and Pfm-inoculated plants, and is regarded as a strategy used by the pathogen to antagonize plant defense mechanisms (Cellini et al., 2014; Nunes da Silva et al., 2019). In fact, several studies have confirmed that *Actinidia* defense responses against Psa are mainly regulated by SA-mediated pathways, whereas ET- and JA-pathways act synergistically between each other, but antagonistically to SA, leading to increased plant susceptibility (Petriccione et al., 2013; Reglinski et al., 2013; Cellini et al., 2014; Petriccione et al., 2014). In the current work, within each species a similar trend in *LOX1* and *SAM* expression was observed, with little variation between Psa- and Pfm-inoculated plants (**Figure 5**). However, it is interesting to note that these genes involved in JA- and ET-pathways were overexpressed (compared to mock-inoculated controls) shortly after infection and decreased thereafter in *A. arguta*, whereas in *A. chinensis* their transcriptional levels remained near the basal threshold (1-fold) during the first stages of the disease, significantly increasing until the end of the experimental period. Due to their reported antagonistic effect to kiwifruit defense mechanisms, we hypothesize that *LOX1* and *SAM* were upregulated in the latter stages of the disease in *A. chinensis* as result of impaired defense ability due to elevated bacterial density inside plant tissues, but were supressed in *A. arguta* as part of its coping mechanisms against Psa and Pfm. The fact that *A. arguta* is able to repress the occurrence of severe impairments to both primary and secondary metabolism from an early stage of the disease can partly explain its increased tolerance to Psa and Pfm infection.

### Plant Antioxidant System Is Activated Earlier in *A. arguta* Than in *A. chinensis*

Increased ROS activity in plant tissues during stress conditions induces lipid peroxidation of cell membranes and organelles, thus leading to the formation of MDA. As such, this metabolite is often used to assess the degree of plant oxidative stress and has been shown to increase following chlorophyll loss in genotypes susceptible to several environmental stresses (Sevengor et al., 2011; Taheri and Kakooee, 2017). Indeed, here we found that lipid peroxidation occurred in Psa- and Pfm-inoculated *A. chinensis* plants 7 dpi, which seems to be in line with the increase of bacterial density inside plant tissues and the decrease of total chlorophylls (**Figure 3**). This demonstrates that inoculation had a negative effect on *A. chinensis* cellular homeostasis, leading to increased lipid peroxidation. On the contrary, *A. arguta* plants showed lower MDA values, regardless of the bacterial inoculum, without significant changes over the experimental period. This is a reflection of the lower bacterial colonization observed in this plant species, as well as the lower extent of leaf symptoms and impairments of primary and secondary metabolism, corroborating the higher tolerance of this plant species do Psa and Pfm.

To counteract lipid peroxidation and other oxidative stress-related impairments, kiwifruit plant responses against Psa include the expression of several genes related with the antioxidant system (Petriccione et al., 2014; Wurms et al., 2017a; Wang et al., 2018; Nunes da Silva et al., 2019). In a previous short-term evaluation, *APX* and *CAT* transcriptional levels in both *A. chinensis* and *A. arguta* were found to be little affected during the first 5 days after Psa and Pfm infection, whereas *SOD* overexpression in *A. chinensis* was observed 5 dpi (Nunes da Silva et al., 2019). Similar results were observed in two-years-old pot-cultivated *A. chinensis* var. *deliciosa* cv. ‘Hayward’ plants inoculated with Psa (Petriccione et al., 2015). In the current work, in *A. chinensis* *SOD* and *APX* regulation seemed to accompany the development of bacterial density (independently of the bacterial strain) and MDA content in plant tissues, significantly increasing when compared with mock-inoculated controls throughout the experimental period (**Figure 4**). Contrastingly, *CAT* upregulation preceded the moments of more intensive bacterial increase and MDA accumulation, probably as a coping strategy against the pathogens. It seems that the activation of plant antioxidant mechanism occurs in a more precocious stage after infection in *A. arguta* and is not dependent on the bacterial density inside plant tissues nor on the extent of MDA accumulation, as *A. arguta* showed lower endophytic bacterial population and MDA concentrations, but still activated its antioxidant system to a higher extent comparing with *A. chinensis*.

### *A. arguta* Seems to Identify and Respond to Psa- and Pfm-Infection From an Earlier Stage After Plant Infection

As part of their defense mechanisms, plants are able to perceive microbe associated molecular patterns, such as flagellin, through pattern recognition receptors (PRR) (Clay et al., 2009). In tomato plants, the resistance protein Pto interacts with *P. syringae* pv. *tomato* effector AvrPto inside plant cells, activating a cascade of defense-related mechanisms (Mucyn et al., 2006). In plants lacking this protein, AvrPto seems to inhibit pathogen recognition and enhance bacterial virulence (Hauck et al., 2003). Genes involved in this process, such as *FLS2* and *CC-NBS-LRR*, were found to be upregulated in *A. chinensis* plants 2 dpi with Psa (Wurms et al., 2017b). In the present work *Pto3* relative fold of expression was increased already at 2 dpi in *A. arguta*, but only at 14 dpi in *A. chinensis* Psa-inoculated plants (**Figure 5**). It seems that in *A. chinensis* *Pto3* expression in Psa-inoculated plants accompanies the increase of endophytic bacterial density, whereas in *A. arguta* it precedes the period of higher bacterial multiplication. Pathogen recognition in the early stages of infection in *A. arguta* plants may explain the earlier activation of the antioxidant system and repression of ET and JA pathways, conferring increased tolerance against Psa and Pfm. Notwithstanding, pathogen recognition mechanisms are highly dependent not only on plant species but also on the type of pathogen. Perhaps the lack of alterations in *Pto3* relative expression in Pfm-inoculated *A. chinensis* plants is due to the activation of other PRR, such as *FLS2* and *CC-NBS-LRR*, supporting the evidence that this class of genes underwent adaptive evolution in response to the corresponding evolution of pathogen avirulence genes (Dangl and Jones, 2001). In fact, genome sequencing revealed a large number of differences between alleles associated with the *avrE1* effector gene between Psa biovar 3 and Pfm (Chapman et al., 2012).

The expression of *Pto3* shortly after *A. arguta* infection with Psa may have allowed the prompt recognition of the pathogen and underpin the higher tolerance of this species. These results also highlight that there is probably a vast repertoire of pathogen recognition genes mediating Actinidia and Psa/Pfm interaction yet to be known, hindering the full understanding of how plants are able to recognize and mitigate noxious pathogens.

## Conclusion

The identification and characterisation of tolerance mechanisms against Psa within *Actinidia* germplasm is of extreme importance for the development of kiwifruit cultivars tolerant to the pathogen. The present work confirmed the greater tolerance of *A. arguta* var. *arguta* to Psa and Pfm infection, compared to *A. chinensis* var. *deliciosa*, since the first had lower bacterial density, higher total chlorophyll concentration and lower MDA concentration throughout the 21 days of experimental period. Moreover, it showed that the higher tolerance of *A. arguta* to Psa and Pfm seems to be related with an early pathogen recognition, with gene *Pto3* being upregulated from an early moment after plant infection, leading to the activation of plant antioxidant system (namely regarding *SOD*, *APX* and *CAT* transcriptional levels) and to the suppression of genes related with ET and JA pathways shortly after infection. The present study analyses a relevant number and variety of parameters related to the dynamics of kiwifruit plants’ response to and Psa and Pfm, contributing to a better understanding of the underlying processes behind differential tolerance against these pathogens. As future works, it would be interesting to perform a broader transcriptomic analysis of *A. chinensis* and *A. arguta* plants inoculated with both Psa and Pfm, as well as the analysis of metabolomic changes that may fully explain the higher tolerance of *A. arguta* to KBC.

## Data Availability Statement

The datasets generated for this study are available on request to the corresponding author.

## Author Contributions

MV and SC designed the study. MG micropropagated and maintained the plants and GB and AM provided the bacterial strains. MN and MG conducted the experimental work, analytical procedures and results analysis. SC coordinated the FCT project that provided the funding for the experiments. All authors contributed to the article and approved the submitted version.

## Funding

This work was supported by National Funds from FCT - Foundation for Science and Technology - through the projects (PTDC/AGR-PRO/6156/2014, UIDB/05748/2020, UIDP/05748/2020, UID/Multi/50016/2019), and through the PhD scholarship from MNS (SFRH/BD/99853/2014).

## Conflict of Interest

The authors declare that the research was conducted in the absence of any commercial or financial relationships that could be construed as a potential conflict of interest.
